# Whole-genome sequencing reveals highly specific gene targeting by in vitro assembled Cas9-ribonucleoprotein complexes in *Aspergillus fumigatus*

**DOI:** 10.1186/s40694-018-0057-2

**Published:** 2018-06-05

**Authors:** Qusai Al Abdallah, Ana Camila Oliveira Souza, Adela Martin-Vicente, Wenbo Ge, Jarrod R. Fortwendel

**Affiliations:** Department of Clinical Pharmacy and Translational Science, University of Tennessee Health Science Center, Memphis, TN USA

**Keywords:** *Aspergillus fumigatus*, CRISPR/Cas9, Genome editing, Off-target mutation

## Abstract

**Background:**

CRISPR/Cas9-based genome editing is quickly becoming a powerful tool within the field of fungal genetics. Adaptation of CRISPR/Cas9 systems are allowing for rapid and highly efficient gene targeting within fungi. We recently reported the adaptation of a simple CRISPR/Cas9 system for gene deletion that is effective across multiple genetic backgrounds of *Aspergillus fumigatus*. This system employs in vitro assembly of Cas9 ribonucleoproteins (RNPs) coupled with micro-homology repair templates for gene deletion. Although highly efficient at gene targeting in wild type genetic backgrounds of *A. fumigatus*, the potential for our system to produce unwanted off-target mutations has not been addressed.

**Results:**

Next-generation Illumina sequencing was used to identify genome mutations among transformants isolated from standard (no Cas9) and Cas9-mediated integration of a hygromycin deletion cassette. Two different concentrations of Cas9 were utilized to examine the association of Cas9 concentration with total numbers and types of genomic mutations. For each of the three test groups (zero, low, and high Cas9), three transformants were sequenced and compared to the parent strain. Bioinformatics analyses revealed the average number of total mutations to be similar among all three test groups. *A. fumigatus* transformation using standard, non-Cas9-mediated methods resulted in an average of 373 ± 28 mutations. In comparison, transformation with in vitro assembled Cas9-RNPs using either high (1 µg/µl) or low (0.5 µg/µl) levels of Cas9 resulted in an average of 326 ± 19 and 395 ± 69 mutations, respectively. In all cases, the vast majority of mutations identified were intergenic. No correlation between the amount of Cas9 utilized for transformation and the overall number of mutations was found. Finally, the specific type of mutation introduced during the transformation process was not Cas9-dependent, as both single-nucleotide polymorphisms and insertion/deletion events were not significantly different between the experimental groups.

**Conclusions:**

CRISPR/Cas9-based genome editing in *A. fumigatus* using in vitro assembled RNPs coupled with microhomology templates is a reliable method of gene targeting. This system is highly efficient and is not associated with increased off-target mutations caused by introduction of the Cas9 nuclease.

## Background

Gene deletion in *A. fumigatus* wild type strains is plagued by low homologous recombination rates and typically requires gene-targeting cassettes that contain ≥ 1000 base pairs of homology to be cloned upstream and downstream of a selection marker. The problem of low homologous recombination rates can be circumvented by using *A. fumigatus* strains mutated to have defective non-homologous end joining (NHEJ) DNA repair pathways [1, 2]. Although these strains have increased gene targeting efficiencies, deletion cassettes still require 500–1000 bp regions of flanking homology and the defective NHEJ pathway(s) should be restored to ensure subtle genetic interactions between the targeted and NHEJ loci do not complicate phenotype interpretation. Finally, standard gene deletion methods in either wild type or NHEJ-defective backgrounds also rely on either multi-step cloning or overlap extension PCR techniques to build gene-targeting cassettes.

To improve gene targeting and genome editing in *A. fumigatus*, CRISPR/Cas9 gene editing technology has recently been implemented. In CRISPR-mediated genome editing, an RNA-directed Cas9 DNA nuclease is employed to recognize and cleave specific DNA sequences after forming a ribonucleoprotein (RNP) complex with a guide RNA (gRNA) [3]. This gRNA is a duplex that is composed of a CRISPR RNA (crRNA) and a transactivating CRISPR RNA (tracrRNA) [3]. The crRNA contains a 20-base region designated as the “protospacer”, which guides specific DNA cleavage by binding to the complementary protospacer in the target genome [4]. However, Cas9-mediated DNA cleavage occurs only if the protospacer is followed by an “NGG” protospacer adjacent motif (PAM) in the target genome [5]. Several CRISPR/Cas9 systems have been developed in *Aspergillus* species [6–11]. In most of these organisms, the Cas9 enzyme and gRNA are introduced via expression constructs that are either contained within autonomously replicating plasmid or are integrated into the genome. Those that employ plasmids, control Cas9 activity through the presence or absence of selective agents in the medium for plasmid maintenance whereas those designed for integration typically rely on regulatable promoters. CRISPR/Cas9 systems are becoming ever more sophisticated in *Aspergillus*, as evidenced by recent work showing that highly-efficient marker-free gene editing can be accomplished in *A. nidulans*, *A. niger*, and *A. oryzae* strains defective in NHEJ [11]. In addition, highly efficient multi-site targeting is now possible in each of these *Aspergillus* species [11]. In *A. fumigatus*, the original use of CRISPR/Cas9 involved strains that constitutively expressed Cas9 from an integrated construct [7]. A later iteration in *A. fumigatus* employed autonomously replicating plasmids for Cas9 and gRNA expression and also utilized selectable marker cassettes (“repair templates” in CRISPR/Cas9 terminology) with microhomology regions that are only 35–50 bp in length, showing that efficient gene targeting can be accomplished in *A. fumigatus* with only small regions of DNA homology [12]. Although CRISPR/Cas9 gene targeting appears highly efficient in *A. fumigatus*, the systems in place thus far rely on genetically altering strains to express the required Cas9 nuclease and/or gRNA components or on building DNA-based constructs for expression of these components [7, 12].

We have reported the adaptation of a CRISPR/Cas9 gene editing system that utilizes in vitro assembled Cas9-RNPs coupled with microhomology repair templates [13]. Rather than genetically altering strains to express Cas9 or gRNAs, in vitro assembly relies on generating the Cas9 RNPs in a test tube before introducing them, along with a repair template (if required), into cells prepared for transformation. In this system, the gRNA was formed in vitro by incubating a mixture of equal molar amounts of crRNA and tracrRNA until a complex is formed. The crRNA and tracrRNA are purchased separately and then assembled into a gRNA complex so that the crRNA can be re-designed for each new protospacer the user desires to target. Next, purified Cas9 enzyme was mixed with the crRNA-tracrRNA complexes and incubated to allow for the formation of Cas9 RNPs. Cas9 concentrations of 1 and 0.5 µg/µl were utilized based on optimization experiments in our laboratory. The Cas9 RNP complexes were then used for standard transformation of *A. fumigatus* protoplasts along with 2 μg of hygromycin resistance cassette that is flanked by 35 base pair homology regions as a repair template. The major advantages of this in vitro assembly system are the simplicity (i.e., does not require strain construction) and the potentially portability from strain to strain. Our system generated nearly 100% gene targeting in the ΔakuB^Δ*Ku80*^ mutant, increased gene deletion frequencies in the wild type strain Af293 from the typical ~ 5% up to ~ 74%, and produced gene targeting efficiencies of ~ 90% in a clinical isolate [13]. Although gene targeting was greatly improved by our method, and is generally highly efficient in CRISPR/Cas9 methods developed for other the *Aspergillus* species, the potential for off-target mutations has largely not been addressed. Multiple recent studies have highlighted the potential for CRISPR/Cas9-based gene editing systems to induce unwanted off-target mutations [14–16]. This is typically believed to occur by promiscuous induction of double strand DNA breaks at sites other than the intended protospacer. To ensure the reliability of our in vitro assembly system, we sought to examine the impact of exogenous Cas9 addition during the transformation process on the induction of genomic mutations in *A. fumigatus* using transformants from the studies we previously performed in the Af293 genetic background.

## Results

To examine the potential for off-target mutations induced by the transient presence of gRNAs and the Cas9 nuclease in our system, we completed next-generation whole-genome sequencing on a subset of transformants isolated from our previous study and performed a comparative analysis of the relative numbers and characteristics of genomic mutations. In our previous study, reporting the optimization of in vitro assembled Cas9 RNPs for gene targeting in *A. fumigatus*, we generated multiple transformants from three basic experimental designs. In the first, we utilized the wild type reference isolate, Af293, and performed a transformation using standard protoplasting protocols to ectopically integrate a hygromycin selection cassette, *hygR* [13]. In the second and third experiments, we used Af293 to perform targeted CRISPR/Cas9-mediated integrations of the same *hygR* cassette to delete the coding region of a polyketide synthase, *pksP* [13]. The *pksP* locus was chosen for protocol optimization as it allowed for colony color-based identification of homologous integrations. The major difference between the second and third experiments of our previous study was the use of either high (1 µg/µl) or low (0.5 µg/µl) concentrations of the Cas9 nuclease, respectively. Therefore, the experimental groups for our whole genome analyses in the current study included three isolates from each of these three transformation conditions: no Cas9 (standard transformation protocol), 0.5 µg/µl Cas9 and 1 µg/µl Cas9 (Table 1). As a reference, we also sequenced the parent strain, Af293. *A. fumigatus* Af293 is the genome reference isolate with a well-annotated genome. However, this parent isolate was re-sequenced to account for genomic mutations that may have arisen during the repetitive sub-culturing of this strain in our laboratory. Average genome coverage ranged from 38× to 62× for all isolates (Table 2).

**Table 1:** Strains used in this study

Strain name	Background
Af293	Wild type
NC1	*hygR* (no Cas9)
NC2	*hygR* (no Cas9)
NC3	*hygR* (no Cas9)
HC4	Δ*pksP*-*hygR* (1 µg/µl Cas9)
HC5	Δ*pksP*-*hygR* (1 µg/µl Cas9)
HC6	Δ*pksP*-*hygR* (1 µg/µl Cas9)
LC7	Δ*pksP*-*hygR* (0.5 µg/µl Cas9)
LC8	Δ*pksP*-*hygR* (0.5 µg/µl Cas9)
LC9	Δ*pksP*-*hygR* (0.5 µg/µl Cas9)

For strains names, NC = no Cas9, HC = high Cas9, and LC = low Cas9. Indicated at the right are the concentrations of Cas9 used to produce each mutant strain. *hygR* = hygromycin resistance cassette; *pksP* = polyketide synthase; Δ*pksP*-*hygR* = *pksP* locus replaced by *hygR*

**Table 2:** Cas9-mediated gene deletion is not associated with increased genomic mutations *in A. fumigatus*

	NC1	NC2	NC3	HC4	HC5	HC6	LC7	LC8	LC9
Average coverage	53×	62×	60×	58×	54×	61×	56×	49×	38×
Total mutations	396	342	385	345	326	307	345	366	474
Average total mutations	373 ± 28 SD			326 ± 19 SD (*p* > 0.05)			395 ± 69 SD (*p* > 0.05)		
*Analysis based on type of mutation identified*									
SNPs	371	318	363	321	314	292	326	347	439
Indels	25	24	22	24	12	15	19	19	35
*Analysis based on location of mutation*									
Intergenic	380 (96%)	331 (97%)	379 (98%)	331 (96%)	320 (98%)	301 (98%)	339 (98%)	359 (98%)	446 (94%)
Average intergenic	363 ± 28 SD			317 ± 15 SD (*p* = 0.04)			381 ± 57 SD (*p* > 0.05)		
Coding region	16 (4%)	11 (3%)	6 (2%)	14 (4%)	6 (2%)	6 (2%)	6 (2%)	7 (2%)	28 (6%)
Average coding region	11 ± 5 SD			9 ± 5 SD (*p* > 0.05)			14 ± 12 SD (*p* > 0.05)		

Displayed are the total and average number of mutations among the three experimental groups: no (“NC”—0 µg/µl), low (“LC”—0.5 µg/µl) and high (“HC”—1 µg/µl) levels of Cas9. For the intergenic and coding region mutation rows, the numbers in parentheses represent the percent (%) of total mutations. For the average mutations per group, the mean ± standard deviation (SD) is provided. The Student’s *t* test assuming unequal variance was used for statistical comparisons of the Cas9 (HC or LC) and the no-Cas9 (NC) groups and *p* values are presented

Comparative bioinformatics analyses revealed that multiple genomic mutations were present in the transformants of all strains, regardless of the presence or absence of Cas9. In the experimental group lacking Cas9, a group average of 373 ± 28 mutations were identified (Table 2). Considered alone, this finding demonstrates the potential mutagenic nature of *A. fumigatus* protoplast transformation. This standard transformation protocol may induce intense, albeit temporary, cellular stress as it requires the enzymatic digestion of the cell wall to release membrane-bound protoplasts followed by recovery on an osmotically stabilized agar medium. Transformants from both the high (1 µg/µl) and low (0.5 µg/µl) Cas9 concentration experiments displayed total numbers of genomic mutations similar to the no Cas9 control, with an average of 326 ± 19 and 395 ± 69, respectively (Table 2). Among all transformants, only a small subset of the identified mutations were located within coding regions of the genome, as the vast majority (> 96% for all isolates) were intergenic (Table 2). A single transformant (LC9) from the low Cas9 concentration experiment was found to contain relatively higher levels of total mutations, with a total of 474, and a slightly increased distribution of these mutations into coding regions (~ 5.9% of total compared to an average of ~ 2.6% for all other transformants). However, as this transformant is an isolate from the low Cas9 concentration experiment, this apparent increase in total number of mutations and distribution towards coding regions is not likely associated with the activity of Cas9. Further supporting this assertion, we found no statistically significant difference in the total number of mutations identified among the experimental groups (Table 2). Therefore, our analyses revealed no Cas9 concentration-dependent increase in induction of genomic mutations. The mutations identified in our study are likely induced by the transformation process, including cell wall digestion.

To further examine if the presence of Cas9 during transformation may influence the type of mutation introduced, we analyzed additional characteristics of the identified genomic variations. Those mutations identified within coding regions displayed comparable distributions among the categories defined in Table 3. In general, most mutations were located within the 3′ UTRs of genes regardless of the presence or absence of Cas9. The only discrepancy noted was that all transformants within the high and low concentration Cas9 groups displayed a low number of non-synonymous mutations identified within coding regions, whereas the standard non-Cas9 transformants were free of non-synonymous mutations (Table 3). However, similar to our findings with total numbers of mutations, the low concentration Cas9 transformants contained more non-synonymous mutations (~ 0.8% of total mutations) than those from the high concentration Cas9 experiment (~ 0.5% of total mutations). Thus, we interpret these mutations not as a consequence of Cas9 presence but as variability among isolates resulting from the standard protoplast transformation process.

**Table 3:** Cas9-mediated gene deletion does not cause alterations in the types of coding region mutations in *A. fumigatus*

	0 µg/µl Cas9			1 µg/µl Cas9			0.5 µg/µl Cas9		
	NC1	NC2	NC3	HC4	HC5	HC6	LC7	LC8	LC9
3′ UTR	9	9	2	9	2	–	1	1	10
5′ UTR	3	–	1	2	–	1	1	–	1
Frameshift	1	–	–	1	–	–	–	–	–
Intron	2	2	2	1	1	1	2	3	2
Non-synonymous	–	–	–	1	1	3	2	1	6
Start lost	–	–	–	–	1	–	–	–	–
Synonymous	–	–	–	–	1	1	–	1	9
Splice region	1	–	1	–	–	–	–	1	–

Shown are the numbers and types of identified mutations located within coding regions of transformants within the three experimental groups

**Fig. 1:**
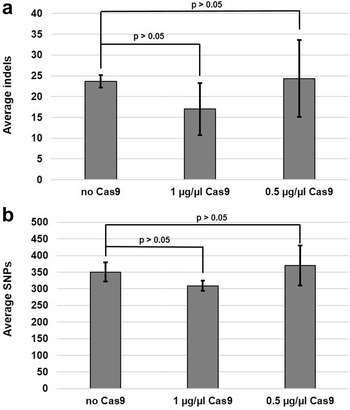
Cas9-mediated gene deletion does not cause a disproportional increase in SNPs or indels in *A. fumigatus*. Segregation of genomic mutations into SNPs (**b**) and indels (**a**) revealed that the concentration of Cas9 was not positively associated with an increase in a specific subset of mutation. Shown are the average number of mutation events within each experimental group. Student’s *t* test assuming unequal variance was utilized for statistical comparison between the “no Cas9” and either the low (0.5 µg/µl) or high (1 µg/µl) Cas9 group

Cas9-induced double strand breaks (DSBs) can be repaired by two major pathways in the cell: the NHEJ DNA repair pathway or homology directed repair [17]. The NHEJ repair pathway is error prone and induces small insertion and deletion (indel) events into the genome [17]. Therefore, a negative consequence of Cas9-mediated gene editing could be the promiscuous induction of DSBs resulting in increased indels throughout the affected transformant(s). To see if this specific type of off-target mutation may exist in our collection, we also analyzed transformants from each experimental group for the disproportional generation of indels versus single nucleotide polymorphisms (SNPs). To do this, the total genomic mutations (intergenic and coding region) were classified into either SNPs or indels and the resulting numbers were averaged for each experimental group. Our data indicated that the generation of SNPs was favored over indels (1–80 nucleotides in length) within each experimental group and no significant differences in the relative amounts of either mutation were noted (Fig. 1). When expressed as a percent of total mutations, the non-Cas9 mediated transformation generated strains containing 6.4% indels, whereas the high and low concentration Cas9 transformants had 5.2 and 6.2% indels, respectively. Therefore, the concentration of Cas9 is not associated with a disproportional increase in the number of indels between experimental groups. Although our protospacers were designed to have minimal off-site complementarity, some level of similarity between our designed sequences and distant areas of the genome is unavoidable. To ensure that off-target complementarity of our gRNA complexes was not promiscuously driving Cas9 to generate unwanted DSBs and subsequent off-target mutations, we finally interrogated each transformant genome for variations surrounding ten potential off-target sites. These off-target sites were defined as areas where the protospacer had twelve or more base pairs of complementarity. For both the 5′ and 3′ protospacer, our analysis found zero genomic mutations in these areas (data not shown). Together, these data indicate that CRISPR/Cas9 editing by our methods is highly specific in *A. fumigatus*.

## Discussion

The implementation of novel gene editing technologies in *A. fumigatus*, like CRISPR/Cas9, is a critical step toward making significant advances in studies related to virulence and antifungal drug resistance in this important human pathogen. We have shown that our system, relying on in vitro assembled Cas9-RNP complexes, produces highly efficient gene targeting in multiple genetic backgrounds of *A. fumigatus* [13]. Because it does not rely on strains that have been genetically engineered to increase homologous recombination rates or to express CRISPR/Cas9 components, this system can be utilized to study gene and pathway function across many isolates. Multiple studies have indicated that CRISPR/Cas9-based gene editing is potentially associated with off-target mutations whereas many studies have also described this system as highly specific [18]. Off-target mutations could be due to promiscuous activity of the Cas9 nuclease, yet more often seem to be caused by non-specific binding of gRNA [18]. Using the system we have adopted for *A. fumigatus*, both of these potential issues are properly addressed [13]. The Cas9 nuclease is introduced as a purified protein and, therefore, is only transiently present. Compared to systems that constitutively or conditionally express Cas9, this should reduce the potential for unwanted mutagenesis of the genome generated by promiscuous Cas9 activity. In fact, in a few yeast and parasite studies, expression of CRISPR/Cas9 components was associated with toxicity [19–21]. Our system avoids this problem entirely. Additionally, using the rules for PAM selection, protospacer design, and RNP assembly that we have previously reported [13], we were able to generate Cas9-transformants that did not show increased genomic variability. Because the genomic variation identified in our study is not correlated with CRISPR/Cas9 methods, our data also suggest that either the single sub-culture of a strain of *A. fumigatus* within the laboratory or the stress induced by our standard protoplast transformation procedure induces genomic variation.

Although whole genome sequencing of transformants has proven useful for the analysis of off-target effects in many systems, this technique does have the limitation of being unable to identify Cas9-induced DSBs that are perfectly repaired. With this caveat in mind, whole genome sequencing has been successfully applied to characterize the potential for off-target effects of Cas9-based transformations in the plant pathogen *Ustilago maydis* [22]. This study relied on expressing Cas9 from a strong constitutive promoter on a self-replicating plasmid. After transformation, strains could then be cured of this plasmid to avoid continuous passage and growth in the presence of Cas9. Whole genome sequencing of transformants acquired in this study revealed that none of the identified genome mutations were likely to be due to Cas9 activity mediated by gRNA binding [22]. Our results support the same conclusion when purified Cas9 is added exogenously to *A. fumigatus*. It is of note, however, that our study only investigated two concentrations of Cas9, did not examine the effects of varying amounts of crRNA or tracrRNA or the ratios of Cas9 to tracrRNA and crRNA, and only investigated off-target effects upon targeting of only one gene. It is possible that by targeting a different protospacer or by significantly altering the Cas9 RNP composition, a different outcome might have been observed. However, even though off-target effects might increase under these other conditions, further efforts that try to minimize them can be pursed. For example, bioinformatic tools are now available to interrogate genomes for potential off-target sites, including Cas-OFFinder and Cas-Designer [23, 24]. Databases like these can aid in protospacer selection and gRNA design to minimize the potential for off-target mutations when targeting a new genomic locus. Also, multiple studies into ways to minimize off-target mutations in CRISPR/Ca9 systems have been published and new techniques are constantly being pursued. One technique might be to limit the time of Cas9 activity in the cell via use of photoactivatable split-Cas9 [25]. Our system accomplishes limited Cas9 activity through introduction of the Cas9 enzyme. However, if required, the specificity of our system could also be further bolstered by employing high-fidelity or rationally engineered Cas9 enzymes with increased specificity or through the use of truncated versions of single-gRNAs [26–28].

## Conclusions

The data provided here demonstrate that CRISPR/Cas9-mediated gene targeting, using our in vitro assembled Cas9-RNP system, does not cause an increase in genomic variation over standard transformation protocols. We also identified no disproportional Cas9-dependent increase in SNPs or indels among treated strains. Therefore, Cas9-mediated gene deletion using in vitro assembled Cas9-RNPs coupled with microhomology repair templates is a reliable method for generating targeted mutations in *A. fumigatus*.

## Methods

### Strains and culture conditions

Strains used for this study are listed in Table 1. Strain Af293 is the *A. fumigatus* reference genome isolate [29] and all transformant strains employed for whole genome sequencing were generated, as part of a previous study, in this genetic background [13]. All strains were maintained on glucose minimal media (GMM) agar [30], supplemented with hygromycin (150 µg/ml) for selection. Conidia were harvested in water from three-day old plates and enumerated by hemocytometer.

### Genomic DNA extraction

Genomic DNA was extracted following a slight modification of previous published protocols, using the Qiagen DNeasy Plant Mini Kit [31]. Briefly, strains were inoculated in GMM broth at a conidial density of 10^6^ conidia/ml and incubated for 20 h at 37 °C with shaking at 250 rpm. Mycelia were harvested by vacuum filtration and 300 mg of a semi-dry mycelial mat was crushed under liquid nitrogen. The resulting mycelial powder was resuspended in 800 μl of buffer AP1 and 8 μl of RNase A and vigorously vortexed. Following 3 h of incubation at 65 °C, the fungal lysate was centrifuged for 5 min at 20,000×*g* and the supernatant was transferred to a new 1.5 ml tube. Next, 260 μl of buffer P3 were added to the supernatant and the mixture was incubated for 5 min on ice, then centrifuged for 5 min at 20,000×*g*. The supernatant was then transferred to QIAshredder spin column (700 μl at a time) and centrifuged for 2 min at 20,000×*g*. The flow-through was collected into a 2 ml tube without disturbing the pellet, and 1.5 volumes of buffer AW1 was added and mixed by pipetting. The mixture was transferred (700 μl at a time) into a DNeasy Mini spin column and the genomic DNA was allowed to bind to the column membrane by centrifuging for 1 min at ≥ 6000×*g*. The genomic DNA was washed first with 700 μl of AW2 buffer and centrifuged for 1 min at ≥ 6000×*g*, followed by a second washing step using 300 μl of Buffer AW2 and centrifugation at 20,000×*g* for 5 min. The second washing step was essential to remove any residual ethanol on the membrane before elution step. The spin column was transferred to a new 1.5 ml tube. For the elution of genomic DNA, 100 μl Buffer 5 mM Tris–HCl (pH 8.5) was added to the center of the column and the column was incubated at room temperature for 5 min, followed by a centrifugation step for 1 min at ≥ 6000×*g*. Final DNA concentrations were quantified using Nanodrop and Qubit Fluorometer, following the manufacturer’s protocol.

### Library preparation and bioinformatics analyses

Library preparations and genome sequencing reactions were performed at the University of Alabama at Birmingham Heflin Center for Genomic Science. The Qiagen QIAseq FX DNA prep kit was used for library preparations, following the manufacturer’s instructions. Paired end 300 base pair sequencing reads were generated on the Illumina MiSeq following standard protocols. Bioinformatics services were provided by code4DNA (www.code4DNA.com). Reads from each sample were aligned using bwa mem (v0.7.15) to the *A. fumigatus* reference genome build A_fumigatus_Af293_version_s03-m05-r05 downloaded from AspGD.org [32]. Samtools (v1.3) fixmate and rmdup were used to remove PCR duplicates [33]. Sequence mutations were called using FreeBayes (v1.1.0) with the haploid population-based model [34]. Low quality (QUAL < 30) and low depth (DP < 10) mutations were filtered out using VCFtools (v0.1.15) which was also used to remove variant calls where no sequence reads were available for at least one sample. The population.vcf file was split into individual samples using VCFtools vcf-subset. Mutations were annotated using snpEff (v4.3r) and VCFtools vcf-isec was used to select mutations found in each affected samples but not in the Ref. [35].

## Abbreviations

CRISPR: clustered regularly interspaced short palindromic repeats
crRNA: CRISPR RNA
DSB: double strand break
GMM: glucose minimal media
gRNA: guide RNA
Indel: insertion/deletion
RNP: ribonucleoprotein
SNP: single-nucleotide polymorphism
tracrRNA: trans-activating crRNA
